# Older Adult Males Who Worked at Small-Sized Workplaces Have an Increased Risk of Decline in Instrumental Activities of Daily Living: A Community-Based Prospective Study

**DOI:** 10.2188/jea.JE20180113

**Published:** 2019-11-05

**Authors:** Kimiko Tomioka, Norio Kurumatani, Keigo Saeki

**Affiliations:** Nara Prefectural Health Research Center, Nara Medical University, Nara, Japan

**Keywords:** active aging, gender differences, instrumental activities of daily living, work history, workplace size

## Abstract

**Background:**

To examine the relationship of working history from early adulthood through old age with instrumental activities of daily living (IADL).

**Methods:**

Analyzed participants were 5,857 community-dwelling older Japanese people aged ≥65 years. Using the Tokyo Metropolitan Institute of Gerontology Index of Competence, IADL decline was defined as individuals who had no IADL dependence at baseline but were deemed as dependent in IADL at follow-up. Work history was based on working status at baseline, total working years, and information concerning the longest held job, including occupation, employment pattern, and workplace size (number of employees). We conducted multiple logistic regression analyses and estimated the odds ratios (ORs) for IADL decline with 95% confidence intervals (CIs) by gender.

**Results:**

At the 33-month follow-up, 428 men (16.6%) and 275 women (8.4%) developed IADL decline. After covariate adjustments, men with unstable employment reported significantly increased IADL decline (OR 1.52; 95% CI, 1.19–1.95) compared to men with stable employment, and men who worked in small workplaces with 1–49 employees had an increased risk for IADL decline (OR 1.53; 95% CI, 1.21–1.93) compared to men in large-sized workplaces with ≥50 employees. After mutual adjustment for all working history items, only the association between small workplaces and IADL decline remained significant in men (OR 1.37; 95% CI, 1.03–1.84). Among women, none of the working history items were associated with IADL decline.

**Conclusion:**

Our results suggest that not only promoting older people’s workforce participation, but also providing workers employed at small workplaces with sufficient occupational health services, may be effective in helping men retain IADL in later life.

## INTRODUCTION

For many countries in the world, including Japan, with its progressive population decline and aging society, prolonging healthy and active life and maintaining the working population are important policy issues.^1^^,^^2^ Regarding workforce participation of older people, there is a perception that older adults who are more vulnerable to physical and cognitive decline are better not to enter the work-force.^3^ Some studies have suggested a positive effect of mandatory retirement on mental health.^4^^,^^5^ However, other studies found that the loss of a job at older age was associated with increased depressive symptoms in community-dwelling older people.^6^^–^^8^ Additionally, late-life working does help older adults succeed in maintaining functional capacity.^7^^,^^9^^–^^13^ Because work can provide workers with a social role, personal fulfillment, and human social ties, which are key mechanisms positively affecting functional capacity, engagement in work may prevent older individuals from losing a purpose in life and withdrawing from the world.^6^^,^^8^ Negative psychological status^14^ and lack of social contact^15^ are significant risk factors for functional decline. Additionally, because old-age working can serve to stimulate the functioning of both body and mind, it may contribute to the prevention of age-related functional decline.^10^^,^^12^

Recently, some researchers have reported that midlife job profiles based on work demands,^16^ working hours in midlife,^17^ and employment experiences before statutory retirement age^18^ can predict functional disability in later life. These findings have indicated that midlife occupational activities have a long-term effect and may influence functional capacity in older age. Although current evidence suggests that occupations during adult life may modify the association between working in later life and old-age functional capacity, few studies have investigated the relationship between working status in older age, employment experiences in adulthood, and functional capacity in later life. Moreover, although there are gender distinctions in employment status, job description, and risk factors for functional decline,^8^^,^^11^^,^^12^^,^^15^ many studies have not performed analyses stratified by gender.^5^^–^^7^^,^^9^^,^^13^^,^^17^^–^^20^ Examining the gender-specific association between old-age working, middle-aged occupation, and functional capability in later life is crucial in addressing the issues of labor shortages and the achievement of a healthy and long-lived society.

This study’s aim is to examine the association between the lifetime work history of community-dwelling aged people, which means employment experiences from early adulthood through old age, and functional capacity, which means the ability to maintain independent living,^21^ for each gender.

## METHODS

### Study participants

Data was obtained through a prospective cohort study conducted by A City in Nara Prefecture, Japan. A City is a typical bedroom community located next to the Osaka metropolitan area, and has a lower percentage of older adults than the national average. For example, as of October 1, 2015, the proportion among the total population of people aged 65 and over was 26.6% in Japan but only 22.3% in A City.

Potentially eligible participants were all older adults who took up residence in A City. The inclusion criteria were people who met all the following three conditions: 1) aged 65 years or older as of January 1, 2014, 2) individuals who lived at home, and 3) individuals who had independent IADL at baseline. The exclusion criteria included people who met at least one of the following conditions: 1) individuals living in a nursing home or individuals in the hospital at survey, 2) individuals with IADL dependence at baseline, and 3) individuals with missing data regarding IADL and/or working history. We distributed self-administered postal questionnaires to all residents aged 65 years and older in March of 2014, and conducted a similar postal questionnaire survey to track participants in November 2016; the follow-up period was 2 years and 9 months (ie, 33 months).

This study was approved by the Nara Medical University Ethics Committee (approval number 939). The submission of self-administered questionnaires was considered agreement to participate in the research.

### Measures

#### Functional capacity

For assessment of functional capacity in community-dwelling older adults, we adopted instrumental activities of daily living (IADL), because ability in IADL is vital for independent living in the community,^22^ and IADL decline can predict future need for nursing care.^23^ IADL at baseline and follow-up was evaluated using five items from the Tokyo Metropolitan Institute of Gerontology Index of Competence (TMIG-IC) with well-established validity and reliability.^24^ The TMIG-IC is a self-report questionnaire measuring participants’ competence to carry out the following five items: using public transportation systems, shopping, preparing meals, paying bills, and handling money. The response to each item is either “practicable” (1 point) or “unfeasible” (0 points). Subjects who obtain a perfect score of 5 points are classified as having IADL independence, while those whose total score is 4 or less are classified as having IADL dependence.^25^^,^^26^ We defined persons with IADL decline as individuals whose IADL score was 5 at baseline but who scored less than that at the 33-month follow-up.^26^

#### Work history

All subjects were asked about their current employment conditions and whether they had had prior work experience. Using the answers to those two questions, working status at baseline was classified into the following three categories: retired (ie, persons who were jobless at baseline, but had worked in the past), working (ie, persons who were engaged in paid work at baseline), and inexperienced (ie, persons who had no history of work experience).^8^ Then, to those who answered that they had worked, we inquired about the total number of years that they had worked (hereafter, total working years). Regarding their longest held job, we enquired about what they did at this job (hereafter, occupation), their employment pattern, and the number of employees in their business establishment (hereafter, workplace size).

Occupation was grouped into 12 classes according to the Japanese Standard Classification of Occupation (JSCO): managers; professionals & technicians; clerical workers; sales workers; service workers; manufacturing workers; transport and machine workers; construction and mining workers; protective services workers; agricultural, forestry, and fishery workers; carrying, cleaning, and packing workers; and others (ie, other unclassified occupations). The validity of the JSCO has been confirmed as a theory-based classification system suitable for measuring occupation-related social position in Japan.^27^ Based on this classification, occupations were re-sorted into white-collar (ie, managers, professionals, and technicians), pink-collar (ie, clerical, sales, and services workers), blue-collar (ie, manufacturing, transport, machine, construction, mining, protective services, agricultural, forestry, fishery, carrying, cleaning, and packing workers), and others.^13^

For employment pattern, we first set the following five choices: fulltime private-sector workers, civil servants, contract/temporary/part-time employees (hereafter, non-regular employees), self-employed/freelancers, and others (ie, other unclassified employment pattern).^28^ Then, employment pattern was categorized as stable (ie, fulltime private-sector workers and civil servants) and unstable (ie, non-regular employees, self-employed/freelancers, and others).

For workplace size, participants were asked to choose one from the following four categories: ≥300 employees, 50–299 employees, 10–49 employees, and 1–9 employees. Finally, workplace size was categorized as large (ie, ≥50 employees) or small (ie, 1–49 employees).^29^

#### Covariates

Based on previous studies,^7^^,^^9^^–^^12^^,^^15^^–^^20^^,^^30^ age, socio-economic status (ie, marital status, education, and self-perceived economic status), health status (ie, body mass index [BMI] and chronic illnesses), lifestyle habits (ie, smoking history, alcohol consumption, and sports habits), and mental function (ie, cognitive functioning and depression) were selected as covariates that may mediate the association between work history and IADL in community-dwelling older adults.

Cognitive functioning was measured using the Cognitive Performance Scale.^31^ The Cognitive Performance Scale has a total score range of 0 to 6, with higher scores expressing a lower level of cognitive functioning. We defined a score of 0 as individuals with intact cognitive functioning and scores of ≥1 as individuals with poor cognitive functioning. Depression was measured with the five-item short form of the Geriatric Depression Scale.^32^ Regarding the five-item version of the Geriatric Depression Scale, the total score ranges from 0 to 5, with higher scores expressing a higher level of depression. We defined scores of <2 as individuals with no depression, and scores of ≥2 as individuals with depression.

Information about gender and age was provided by the municipality, and data on other covariates was drawn from the questionnaire. For missing covariate values, in accordance with a statistical method for the purpose of dealing with missing covariates,^33^ multiple imputations by chained equations were implemented. Details of the covariates excluding mental function and multiple imputations are described in the supplementary data (eAppendix 1), and characteristics of study participants by gender are shown in eTable 1.

### Statistical analyses

Multiple logistic regression models were applied to estimate adjusted odds ratio (OR) for IADL decline and a 95% confidence interval (CI). The independent variable was working history. In model 1, the age-adjusted OR was calculated. In model 2, all covariates (ie, age, socio-economic status, health status, lifestyle habits, and mental functioning) were added simultaneously, and the multivariable-adjusted OR was calculated. The analyses performed were stratified by gender.

The level of significance was 0.05 (two-tailed). IBM SPSS Statistics (version 24.0; IBM Corp, Armonk, NY, USA) was used for statistical analyses.

## RESULTS

**Figure 1.**
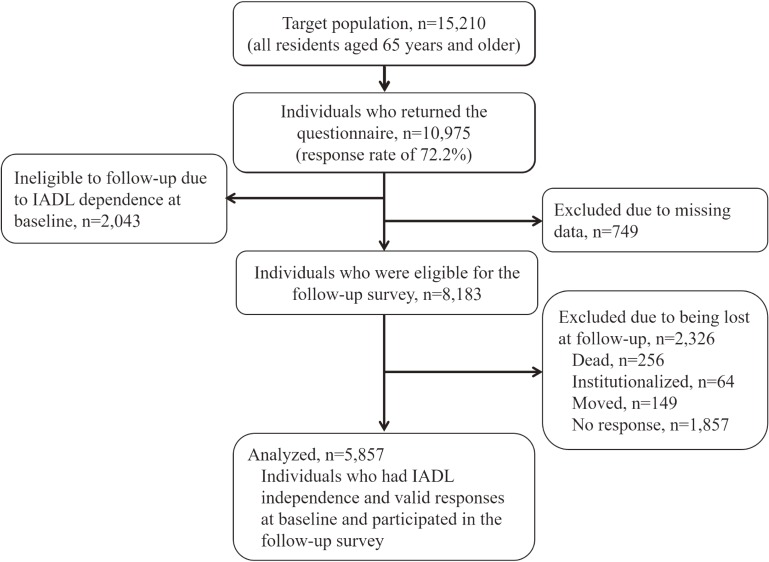
Flow chart of the enrollment of study participants. IADL, instrumental activity of daily living.

Among the target population (*n* = 15,210), 10,975 returned the questionnaires (response rate 72.2%). Among the responses, 8,183 persons were eligible for the follow-up survey. We failed to track 2,326 persons due to death (*n* = 256), institutionalization (*n* = 64), relocation (*n* = 149), or non-response (*n* = 1,857). The final analyses were conducted for 5,857 individuals (Figure 1). Excluded persons were more likely to be older, have poor cognitive performance, and be afflicted with depression than those included. However, there was no gender difference between the two groups (Table 1).

**Table 1.** Basic attributes of analyzed participants and excluded individuals

	Analyzed subjects (*n* = 5,857)	Excluded subjects^a^ (*n* = 3,075)	*P*^b^
Age at baseline			<0.001
65–74 years	3,909 (66.7)	1,695 (55.1)	
75 years or older	1,948 (33.3)	1,380 (44.9)	
Gender			0.893
Male	2,572 (43.9)	1,345 (43.7)	
Female	3,285 (56.1)	1,730 (56.3)	
Cognitive functioning^c^ at baseline			<0.001
Intact	4,922 (84.0)	2,345 (76.3)	
Poor	843 (14.4)	559 (18.2)	
Missing	92 (1.6)	171 (5.6)	
Depression^d^ at baseline			<0.001
No depression	4,385 (74.9)	1,955 (63.6)	
Depression	1,158 (19.8)	750 (24.4)	
Missing	314 (5.4)	370 (12.0)	

Data are given as *n* (%).

^a^Individuals who were excluded because of missing data (*n* = 749) or being lost at follow-up (*n* = 2,326).

^b^Chi-squared test.

^c^Evaluated using the Cognitive Performance Scale.

^d^Evaluated using the five-item short form of the Geriatric Depression Scale.

Of the analyzed subjects, their age at baseline averaged 72.7 (standard deviation, 5.8) years, 43.9% were male, and 6.9% reported having no work experience. At the 33-month follow-up, 428 men (16.6%) and 275 women (8.4%) exhibited IADL decline. Men were more likely to be married, have higher educational background, have more chronic illnesses, smoke and drink more, be less active in sports, and have poorer cognition than women. Women were more likely to have low BMI and depression than men. Age and self-perceived economic status did not differ between the genders (Table 2).

**Table 2.** Baseline characteristics of the study population by gender (*n* = 5,857)

Baseline characteristics	Men (*n* = 2,572)	Women (*n* = 3,285)	*P*^a^
	*n* (%)	*n* (%)	
Age, mean (SD) years	72.7 (5.7)	72.7 (5.9)	0.737
Marital status: married	2,282 (88.7)	2,116 (64.4)	<0.001
Education: junior college or higher	823 (32.0)	581 (17.7)	<0.001
Self-perceived economic status: very poor	359 (14.0)	517 (15.7)	0.060
Body mass index, mean (SD) kg/m^2^	23.1 (2.7)	22.5 (3.2)	<0.001
Number of chronic illnesses^b^ ≥2	583 (22.7)	549 (16.7)	<0.001
Former or current smokers	1,818 (70.7)	266 (8.1)	<0.001
Daily/occasional drinkers	1,130 (43.9)	214 (6.5)	<0.001
Subjects regularly participating in sports^c^	557 (21.7)	905 (27.5)	<0.001
Subjects with poor cognitive functioning^d^	404 (15.7)	439 (13.4)	0.012
Subjects with depression^e^	433 (16.8)	725 (22.1)	<0.001

SD, standard deviation.

^a^*P*-values from chi-squared tests for categorical variables and *t*-test for continuous variables.

^b^Chronic illnesses include hypertension, diabetes mellitus, heart disease, cerebrovascular disease, and ophthalmic disorder.

^c^Subjects who exercised once a week or more.

^d^Cognitive functioning was evaluated using the Cognitive Performance Scale (range 0–6), and poor cognitive functioning was defined as a score of 1 or greater.

^e^Depression was evaluated using the 5-item short form of the Geriatric Depression Scale (range 0–5), and the presence of depression was defined as a score of 2 or greater.

Regarding working status at baseline, more men (29.0%) had paid work than women (14.8%), while more women (10.2%) had no work experience than men (2.7%). Regarding total working years, the proportion of men with ≥25 years was 93.0%, while that of females was 36.5%. For longest held jobs, men tended to engage in white-collar jobs (47.1%), have stable employment (75.1%), and belong to large-sized workplaces (65.9%), while most women held pink-collar positions (48.7%), followed an unstable employment path (51.5%), and worked in small workplaces (51.3%).

The detailed results, including crude ORs, are shown in eTable 2 and eTable 3. Table 3 shows the adjusted ORs of key work history indicators. In both men and women, neither working status at baseline nor total working years were associated with IADL decline. For occupation, among both genders, participants engaged in blue-collar jobs tended to have an increased OR for IADL decline compared to white-collar workers, but these trends were not observed after adjustment for all covariates. For employment pattern, men with an unstable employment history had a significantly higher OR for IADL decline than men with stable employment, even after adjustment for all covariates (OR 1.52; 95% CI, 1.19–1.95). In contrast, among women, employment pattern was not linked to IADL decline. Interaction terms between gender and employment pattern did not have statistical significance (*P* for interaction = 0.369). For workplace size, after age adjustment, compared to large-sized workplaces, small workplaces were significantly associated with IADL decline among both genders. However, after adjustment for all covariates, significant association was observed only in men: men who worked at small workplaces had a significantly higher OR for IADL decline than those who worked at large-sized workplaces (OR 1.53; 95% CI, 1.21–1.93). Interaction terms between gender and workplace size were not significant (*P* for interaction = 0.788).

**Table 3.** Adjusted odds ratios for decline in instrumental activities of daily living by gender

	Men (*n* = 2,572)		Women (*n* = 3,285)	
	
	Model 1	Model 2	Model 1	Model 2
	Age-adjusted OR (95% CI)	Multivariable-adjusted OR^a^ (95% CI)	Age-adjusted OR (95% CI)	Multivariable-adjusted OR^a^ (95% CI)
Working status at baseline				
Retired	1.00	1.00	1.00	1.00
Working	0.94 (0.73–1.21)	0.96 (0.74–1.24)	0.63 (0.37–1.06)^†^	0.71 (0.41–1.22)
Inexperienced	1.25 (0.70–2.24)	1.28 (0.71–2.31)	1.10 (0.75–1.61)	1.18 (0.79–1.76)
Total working years				
≥25	1.00	1.00	1.00	1.00
1–24	1.26 (0.79–2.02)	1.22 (0.76–1.97)	1.13 (0.84–1.50)	1.11 (0.82–1.51)
0 (inexperienced)	1.29 (0.72–2.29)	1.30 (0.72–2.35)	1.23 (0.80–1.87)	1.29 (0.83–2.01)
Occupation for the longest held job				
White collar^b^	1.00	1.00	1.00	1.00
Pink collar^c^	1.07 (0.82–1.39)	1.06 (0.80–1.38)	1.09 (0.71–1.67)	0.91 (0.57–1.45)
Blue collar^d^	1.30 (0.99–1.69)^†^	1.19 (0.89–1.60)	1.67 (1.05–2.64)^*^	1.18 (0.72–1.94)
Others	0.82 (0.41–1.64)	0.73 (0.36–1.49)	1.68 (0.96–2.96)^†^	1.06 (0.58–1.94)
Inexperienced	1.37 (0.76–2.47)	1.35 (0.74–2.47)	1.44 (0.86–2.41)	1.21 (0.70–2.10)
Employment pattern for the longest held job				
Stable^e^	1.00	1.00	1.00	1.00
Unstable^f^	1.56 (1.23–1.98)^*^	1.52 (1.19–1.95)^*^	1.22 (0.92–1.64)	1.08 (0.80–1.46)
Inexperienced	1.44 (0.81–2.58)	1.45 (0.80–2.63)	1.29 (0.84–1.96)	1.27 (0.82–1.96)
Workplace size for the longest held job				
Large (≥50 employees)	1.00	1.00	1.00	1.00
Small (1–49 employees)	1.57 (1.26–1.96)^*^	1.53 (1.21–1.93)^*^	1.39 (1.03–1.88)^*^	1.28 (0.94–1.75)
Inexperienced	1.50 (0.84–2.70)	1.52 (0.83–2.75)	1.41 (0.92–2.16)	1.42 (0.90–2.22)

CI, confidence interval; OR, odds ratio.

^a^Adjusted for age, marital status, education, self-perceived economic status, body mass index, chronic illnesses, smoking, alcohol consumption, sports habits, cognitive functioning, and depression.

^b^White collar: managers, professionals, and technicians.

^c^Pink collar: clerical, sales, and services workers.

^d^Blue collar: manufacturing, transport, machine, construction, mining, protective services, agricultural, forestry, fishery, carrying, cleaning, and packaging workers.

^e^Stable: full-time private sector workers and civil servants.

^f^Unstable: non-regular employees, self-employed/freelancers, and others.

^†^*P* < 0.10, ^*^P < 0.05.

After confirming that there were no problems with multicollinearity (results are shown in eTable 4), we constructed a mutually adjusted model for all items of working history to evaluate the independent effects of each working history item on IADL decline (Table 4). Among men, unstable employment lost its significance (OR 1.33; 95% CI, 0.97–1.84), while the association between small workplaces and IADL decline remained significant after mutual adjustments (OR 1.37; 95% CI, 1.03–1.84), suggesting that workplace size is an independent predictor of IADL decline among male older adults.

**Table 4.** Adjusted odds ratios for decline in instrumental activities of daily living: mutually adjusted model for all items of working history.

	Men (*n* = 2,572) OR^a^ (95% CI)	Women (*n* = 3,285) OR^a^ (95% CI)
Working status at baseline (reference: retired)		
Working	0.86 (0.66–1.12)	0.70 (0.40–1.22)
Inexperienced	1.54 (0.84–2.82)	1.34 (0.73–2.46)
Total working years (reference: ≥25 years)		
1–24 years	1.08 (0.66–1.76)	1.10 (0.80–1.50)
Occupation for the longest held job (reference: white collar^b^)		
Pink collar^c^	1.00 (0.76–1.31)	0.86 (0.54–1.37)
Blue collar^d^	1.08 (0.80–1.46)	1.09 (0.66–1.82)
Others	0.53 (0.25–1.10)^†^	0.96 (0.52–1.79)
Employment pattern for the longest held job (reference: stable^e^)		
Unstable^f^	1.33 (0.97–1.84)^†^	0.92 (0.64–1.32)
Workplace size for the longest held job (reference: large^g^)		
Small^h^	1.37 (1.03–1.84)^*^	1.34 (0.93–1.93)

CI, confidence interval; OR, odds ratio.

^a^Adjusted for age, marital status, education, self-perceived economic status, body mass index, chronic illnesses, smoking, alcohol consumption, sports habits, cognitive functioning, depression, working status at baseline, total working years, occupation, employment pattern, and workplace size.

^b^White collar: managers, professionals, and technicians.

^c^Pink collar: clerical, sales, and services workers.

^d^Blue collar: manufacturing, transport, machine, construction, mining, protective services, agricultural, forestry, fishery, carrying, cleaning, and packaging workers.

^e^Stable: full-time private sector workers and civil servants.

^f^Unstable: non-regular employees, self-employed/freelancers, and others.

^g^Large: ≥50 employees.

^h^Small: 1–49 employees.

^†^*P* < 0.10, ^*^P < 0.05.

Additionally, we conducted sensitivity analyses between imputed and complete case datasets (results are shown in eTable 5) and confirmed that the results obtained from complete data were similar to the pattern of those from multiple imputation data.

## DISCUSSION

Among men, independent of their other work history and other aspects of socioeconomic status, the size of workplace of their longest held job was related to their IADL. Men who worked in small workplaces had a significantly higher risk of IADL decline compared to those who worked in larger workplaces. On the other hand, women had no associations between lifetime work history and IADL decline. To our knowledge, this is the first study to investigate the association between working history from early adulthood through old age and IADL among community-dwelling older adults, based on analyses stratified by gender.

Many prior studies reported that health status among workers in small-scale enterprises was worse than those in large-scale companies. For example, the prevalence of hypertension, impaired glucose tolerance, current smoking, physical inactivity, and obesity was higher in small worksites with fewer than 50 workers than in large-sized worksites with 50 workers or more.^34^^–^^36^ Additionally, hypertension, physical inactivity, and obesity in midlife have been identified as predictors of impaired physical functioning in old age,^19^ and the accumulation of cardiovascular risk factors, such as hypertension, hyperlipidemia, hyperglycemia, current smoking, and obesity, can have negative effects on IADL in community-dwelling older adults.^37^ Therefore, male workers engaged in small-sized workplaces may have more health and behavioral problems in midlife than those in large-sized workplaces, leading to a negative effect on IADL performance in old age.

In Japan, under the Industrial Safety and Health Act, business owners are obligated to conduct health checks on their employees once or more per year. However, employers of small businesses with fewer than 50 employees are released from the legal liability of reporting the results of annual medical examinations to the Labor Standards Inspection Office. According to the Survey on the State of Employees’ Health,^38^ the rate of business owners who conducted regular health checks in the past year was 100.0% of workplaces of 300 or more employees, 98.9% of those with 50 to 299 employees, 92.6% of those with 30 to 49 employees, and 82.7 of those with 10 to 29 employees. This shows that the smaller the workplace size, the lower the rate of regular health checks. Furthermore, the rate of workplaces that conducted subsequent measures for employees with abnormal findings was also lower for smaller workplaces.^38^ Because small-scale enterprises are poor in manpower, financial power, and technology access, workers in small-sized companies may be offered a lower-quality occupational health service than those in large companies.^39^ Therefore, taking measures for sufficient provision and quality improvement of health management for male workers in small workplaces with fewer than 50 employees may be an important way of preventing IADL decline in older age.

In this study, female working history had no associations with IADL decline. Our finding disagrees with the findings of previous studies, which showed that women who were working at older age had beneficial effects on IADL,^12^ and that midlife job profiles were associated with disability among female older people.^16^ Regarding possible explanations for inconsistent findings, our previous study^12^ examined the relationship between the continuity of working at older age and the emergence of IADL decline but failed to distinguish women with working experience from the non-working group. Second, Prakash’s study^16^ was targeted at municipal employees aged 44–58 years at baseline; civil servants have permanent and full-time employment. Therefore, the absence of association in our study might be due to the presence of persons without work experience, the shortness of work experience, and a minority of full-time workers.

This study has some strengths. First, because it was a community-based study, our study participants included people without work experience. This approach can succeed in attenuating the healthy worker effect. Additionally, we included workers with lower socioeconomic status, such as private-sector employees, self-employed/freelancers, and non-regular employees, compared to previous studies that focused on workers with higher socioeconomic status, such as civil servants^4^^,^^16^^,^^19^ white-collar workers,^17^ and economically active men.^17^^,^^28^ This may have an advantage in the generalization of community-dwelling old adults. Second, we evaluated not only working status later in life, but also working experiences before older age, by gender.

There are some limitations in this study. First, we did not use the International Standard Classification of Occupations.^40^ Therefore, our study has a limitation in terms of international comparisons to occupational statistics. However, because the JSCO is used in nationally representative surveys and is a well-recognized classification tool in Japan, this may make it easier for older adults to provide an answer and contribute to preventing misclassification or missing data. Second, we should consider the effect of non-response and loss to follow-up. In this study, excluded individuals were significantly older and had poorer mental health than those who were analyzed. Because poor mental functioning as well as advanced age have been reported to predict IADL decline,^9^^,^^26^ the possibility that excluded persons might develop IADL decline is high. This bias may result in a weakening of the association between work history and IADL. Third, our findings were based on self-reported data. This may produce misclassifications in measured data and overestimation between work history and IADL. Finally, our outcome was only IADL decline; we failed to evaluate IADL dynamics. Because previous studies suggest that IADL decline is reversible,^9^^,^^20^ further study is needed to verify whether work history has implications for IADL recovery as well as IADL decline.

In conclusion, we found that older men who had worked for small workplaces were at high risk for IADL decline. Our findings could not be fully explained by other work history and other factors related to socioeconomic status. Although the active utilization of older workers has been promoted, our study suggests that to maintain the IADL of community-dwelling older adults, it may be necessary and effective to endeavor to solve inequalities in work hygiene of workers in small-sized workplaces with fewer than 50 employees. For example, workers in small companies could receive the same level of health management activities as those conducted at large companies.

## APPENDIX A. SUPPLEMENTARY DATA

eAppendix 1. Detailed explanation of covariates and multiple imputations

eTable 1. Characteristics of study participants by gender (n = 5,857)

eTable 2. Odds ratios for decline in instrumental activities of daily living (95% confidence intervals) by work history (n = 2,572 men)

eTable 3. Odds ratios for decline in instrumental activities of daily living (95% confidence intervals) by work history (n = 3,285 women)

eTable 4. Results of consideration for multicollinearity

eTable 5. Sensitivity analyses between imputed and complete case datasets
