# Supplementary material for: Older Adult Males Who Worked at Small-Sized Workplaces Have an Increased Risk of Decline in Instrumental Activities of Daily Living: A Community-Based Prospective Study
Source: J Epidemiol. 2019 Nov 5;29(11):407–13. doi: 10.2188/jea.JE20180113 (PMC6776476; doi:10.2188/jea.JE20180113)
Supplement: Supplementary file 1 [file je-29-407-s001.pdf]

Supplementary data

eAppendix 1. Detailed explanation of covariates and multiple imputations

Covariates

Age was categorized as 65–69, 70–74, 75–79, and ≥80 years. Marital status was categorized as either married or not married. Educational background was categorized as junior college or higher, high school, and junior high school or less. For self-perceived economic status, we asked respondents how they felt about their current financial state of affairs, giving them four answers to choose from: "very rich," "somewhat rich," "somewhat poor," and "very poor". Self-perceived economic status was categorized as very/somewhat rich, somewhat poor, and very poor. Body mass index (kg/m^2) was categorized as normal (18.5 to <25.0), underweight (<18.5), and overweight (≥25.0). Chronic illnesses included hypertension, diabetes mellitus, heart disease, cerebrovascular disease, and ophthalmic disorder, because these five diseases were reported to be associated with instrumental activities of daily living (IADL) decline among community-dwelling older adults.[1] Subjects were asked if they were currently under medical treatment for hypertension, diabetes mellitus, heart disease, cerebrovascular disease, and ophthalmic disorder. Respondents selected "yes" or "no"

for each illness. The number of chronic illnesses under medical treatment was categorized as none, one, and ≥2. Smoking history was categorized as never-smokers and ex/current smokers. Alcohol consumption was categorized as non/social drinkers and daily/occasional drinkers. For sports habits, subjects were asked about frequency of exercise during the past year; “once per week or more,” “several times per month,” “several times per year,” and “almost never”. Subjects who answered ‘‘once per week or more’’ were defined as active, and those who answered “less than once per week” were defined as inactive.[2]

Multiple imputations

Using logistic regression, we created five sets of imputation data and performed analyses on the complete pooled data set. Gender, age, marital status, educational background, self-perceived economic status, body mass index, chronic illnesses, smoking history, alcohol consumption, sports habits, cognitive functioning, depression, all the independent variables (i.e., all items of work history), and outcome (i.e., IADL decline) were entered into the imputation procedure. For this study, there were no missing data on gender and age. Marital status (1.7% missing), educational background (0.9% missing), self-perceived economic status (7.7% missing), body mass index (3.8%

missing), chronic illnesses (9.4% missing), smoking history (5.0% missing), alcohol consumption (4.6% missing), sports habits (1.6% missing), cognitive functioning (1.6% missing), and depression (5.4% missing) were imputed as ordinal variables. Multiple imputations were conducted using the IBM SPSS Missing Values Option.

We compared the individuals with and without missing covariate data. Compared to individuals with perfect covariates, those with missing covariate values were older and more likely to be female; the proportion of people aged 75 or older was 31.1% and 41.0%, and the proportion of women was 54.6% and 61.5%, for individuals with complete data and those with missing data, respectively.

REFERENCES

1. Fujiwara Y, Shinkai S, Kumagai S, et al. Impact of history or onset of chronic medical conditions on higher-level functional capacity among older community-dwelling Japanese adults. Geriatr Gerontol Int. 2003;3:S69–S77.
2. Kanamori S, Kai Y, Kondo K, et al. Participation in sports organizations and the prevention of functional disability in older Japanese: the AGES cohort study. PLoS One 2012;7:e51061.

eTable 1. Characteristics of study participants by gender (n=5,857)

Characteristics	Men (n=2,572)		Women (n=3,285)		P^a
Age at baseline					0.223
65–69 years	873	(33.9)	1,149	(35.0)	
70–74 years	846	(32.9)	1,041	(31.7)	
75–79 years	515	(20.0)	616	(18.8)	
≥80 years	338	(13.1)	479	(14.6)	
**Socio-economic status**					
Marital status					<0.001
Married	2,282	(88.7)	2,116	(64.4)	
Not married	256	(10.0)	1,104	(33.6)	
Missing	34	(1.3)	65	(2.0)	
Educational background					<0.001
Junior college or higher	823	(32.0)	581	(17.7)	
High school	1,160	(45.1)	1,831	(55.7)	
Junior high school or less	572	(22.2)	837	(25.5)	
Missing	17	(0.7)	36	(1.1)	
Self-perceived economic status					0.069
Very/somewhat rich	1,000	(38.9)	1,210	(36.8)	
Somewhat poor	1,031	(40.1)	1,290	(39.3)	
Very poor	359	(14.0)	517	(15.7)	
Missing	182	(7.1)	268	(8.2)	
**Health status**					
Body mass index (kg/m^2)					<0.001
Normal (18.5–24.9)	1,853	(72.0)	2,305	(70.2)	
Underweight (<18.5)	86	(3.3)	265	(8.1)	
Overweight (≥25.0)	545	(21.2)	583	(17.7)	
Missing	88	(3.4)	132	(4.0)	
Number of chronic illnesses[b] under medical treatment					<0.001
None	786	(30.6)	1,125	(34.2)	
One	986	(38.3)	1,275	(38.8)	
≥2	583	(22.7)	549	(16.7)	
Missing	217	(8.4)	336	(10.2)	

eTable 1. Continued.

Characteristics	Men (n=2,572)		Women (n=3,285)		*P*[a]
**Lifestyle habits**					
Smoking history					<0.001
Never-smokers	625	(24.3)	2,854	(86.9)	
Former or current smokers	1,818	(70.7)	266	(8.1)	
Missing	129	(5.0)	165	(5.0)	
Alcohol consumption					<0.001
Non/social drinkers	1,312	(51.0)	2,933	(89.3)	
Daily/occasional drinkers	1,130	(43.9)	214	(6.5)	
Missing	130	(5.1)	138	(4.2)	
Sports habits					<0.001
Active: once per week or more	557	(21.7)	905	(27.5)	
Inactive: less than once per week	1,991	(77.4)	2,312	(70.4)	
Missing	24	(0.9)	68	(2.1)	
**Mental function**					
Cognitive functioning (score of the Cognitive Performance Scale)					0.017
Intact (0)	2,122	(82.5)	2,800	(85.2)	
Poor (≥1)	404	(15.7)	439	(13.4)	
Missing	46	(1.8)	46	(1.4)	
Depression[c] (score per the 5-item short form of the Geriatric Depression Scale)					<0.001
No depression (0-1)	1,991	(77.4)	2,394	(72.9)	
Depression (≥2)	433	(16.8)	725	(22.1)	
Missing	148	(5.8)	166	(5.1)	

eTable 1. Continued.

Characteristics	Men (n=2,572)		Women (n=3,285)		*P*[a]
**Work history**					
Working status at baseline					<0.001
Retired: persons who were jobless at baseline, but had work experience	1,758	(68.4)	2,464	(75.0)	
Working: persons who engaged in paid work at baseline	745	(29.0)	486	(14.8)	
Inexperienced: persons who had no work experience	69	(2.7)	335	(10.2)	
Total working years					<0.001
≥25 years	2,391	(93.0)	1,199	(36.5)	
15–24 years	25	(1.0)	592	(18.0)	
5–14 years	27	(1.0)	767	(23.3)	
1–4 years	60	(2.3)	392	(11.9)	
0 years (Inexperienced)	69	(2.7)	335	(10.2)	
Occupation for the longest held job					<0.001
Managers	597	(23.2)	46	(1.4)	
Professionals & technicians	614	(23.9)	420	(12.8)	
Clerical workers	284	(11.0)	950	(28.9)	
Sales workers	280	(10.9)	311	(9.5)	
Services workers	107	(4.2)	339	(10.3)	
Manufacturing workers	254	(9.9)	579	(17.6)	
Transport and machine workers	105	(4.1)	4	(0.1)	
Construction and mining workers	92	(3.6)	12	(0.4)	
Protective services workers	39	(1.5)	0	(0.0)	
Agricultural, forestry, and fishery workers	33	(1.3)	11	(0.3)	
Carrying, cleaning, and packaging workers	27	(1.0)	73	(2.2)	
Others: other unclassified occupation	71	(2.8)	205	(6.2)	
Inexperienced	69	(2.7)	335	(10.2)	

eTable 1. Continued.

Characteristics	Men (n=2,572)		Women (n=3,285)		*P*[a]
Employment pattern for the longest held job					<0.001
Full-time private sector workers	1,547	(60.1)	997	(30.4)	
Civil servants	384	(14.9)	261	(7.9)	
Non-regular employees (i.e., contract, temporary, part-time employees)	31	(1.2)	774	(23.6)	
Self-employed/freelancers	468	(18.2)	729	(22.2)	
Others	73	(2.8)	189	(5.8)	
Inexperienced	69	(2.7)	335	(10.2)	
Workplace size for the longest held job: number of employees					<0.001
≥300 employees	1,211	(47.1)	703	(21.4)	
50–299 employees	484	(18.8)	562	(17.1)	
10–49 employees	317	(12.3)	637	(19.4)	
1–9 employees	491	(19.1)	1,048	(31.9)	
Inexperienced	69	(2.7)	335	(10.2)	

Data are given as n (%).

[a]Chi-squared test.

[b]Chronic illnesses include hypertension, diabetes mellitus, heart disease, cerebrovascular disease, and ophthalmic disorder.

[c]Depression was evaluated using the 5-item short form of the Geriatric Depression Scale (range 0–5), and the presence of depression was defined as a score of 2 or greater.

eTable 2. Odds ratios for decline in instrumental activities of daily living (95% confidence intervals) by work history (n=2,572 men)

	n	Unadjusted	Model 1[a]	Model 2[b]
Working status at baseline				
Retired	1,758	1.00	1.00	1.00
Working	745	0.75 (0.59-0.96)*	0.94 (0.73-1.21)	0.96 (0.74-1.24)
Inexperienced	69	1.42 (0.80-2.51)	1.25 (0.70-2.24)	1.28 (0.71-2.31)
Total working years				
≥25 years	2,391	1.00	1.00	1.00
15–24 years	25	1.63 (0.65-4.11)	1.44 (0.56-3.68)	1.42 (0.55-3.71)
5–14 years	27	0.90 (0.31-2.61)	0.84 (0.29-2.47)	0.73 (0.25-2.19)
1–4 years	60	1.57 (0.86-2.89)	1.39 (0.75-2.57)	1.40 (0.74-2.62)
0 years (inexperienced)	69	1.56 (0.88-2.76)	1.29 (0.72-2.29)	1.30 (0.72-2.35)
Total working years (relisted)				
≥25 years	2,391	1.00	1.00	1.00
1–24 years	112	1.41 (0.89-2.24)	1.26 (0.79-2.02)	1.22 (0.76-1.97)
0 years (inexperienced)	69	1.56 (0.88-2.76)	1.29 (0.72-2.29)	1.30 (0.72-2.35)
Occupation for the longest held job				
Managers	597	1.00	1.00	1.00
Professionals & technicians	614	0.98 (0.72-1.34)	1.02 (0.74-1.40)	0.93 (0.67-1.30)
Clerical workers	284	0.86 (0.58-1.29)	0.88 (0.58-1.32)	0.85 (0.56-1.30)
Sales workers	280	1.12 (0.77-1.64)	1.23 (0.84-1.81)	1.15 (0.77-1.72)
Services workers	107	1.17 (0.68-2.01)	1.29 (0.75-2.24)	1.13 (0.64-2.01)
Manufacturing workers	254	1.43 (0.98-2.08)†	1.39 (0.95-2.03)†	1.20 (0.79-1.82)
Transport and machine workers	105	1.69 (1.03-2.79)*	1.70 (1.02-2.83)*	1.54 (0.89-2.66)
Construction and mining workers	92	1.06 (0.58-1.92)	1.02 (0.56-1.87)	0.86 (0.46-1.63)
Protective services workers	39	0.80 (0.30-2.09)	0.89 (0.34-2.36)	0.90 (0.33-2.45)
Agricultural, forestry, and fishery workers	33	1.73 (0.76-3.96)	1.44 (0.62-3.34)	1.48 (0.61-3.57)
Carrying, cleaning, and packaging workers	27	0.68 (0.20-2.30)	0.68 (0.20-2.32)	0.52 (0.15-1.82)
Others	71	0.89 (0.44-1.80)	0.83 (0.41-1.69)	0.71 (0.34-1.47)
Inexperienced	69	1.64 (0.90-2.99)	1.38 (0.75-2.54)	1.31 (0.70-2.45)
Occupation (relisted)				
White collar[c]	1,211	1.00	1.00	1.00
Pink collar[d]	671	1.03 (0.79-1.33)	1.07 (0.82-1.39)	1.06 (0.80-1.38)
Blue collar[e]	550	1.35 (1.04-1.76)*	1.30 (0.99-1.69)†	1.19 (0.89-1.60)
Others	71	0.90 (0.45-1.78)	0.82 (0.41-1.64)	0.73 (0.36-1.49)
Inexperienced	69	1.65 (0.93-2.95)†	1.37 (0.76-2.47)	1.35 (0.74-2.47)

eTable 2. Continued

	n	Unadjusted	Model 1[a]	Model 2[b]
Employment pattern for the longest held job				
Full-time private sector workers	1,547	1.00	1.00	1.00
Civil servants	384	0.87 (0.63-1.21)	0.83 (0.60-1.16)	0.82 (0.59-1.15)
Non-regular employees	31	1.66 (0.71-3.90)	1.70 (0.72-4.03)	1.64 (0.68-3.96)
Self-employed/freelancers	468	1.77 (1.37-2.29)*	1.60 (1.23-2.07)*	1.56 (1.19-2.04)*
Others	73	1.12 (0.59-2.11)	0.93 (0.49-1.77)	0.90 (0.47-1.74)
Inexperienced	69	1.72 (0.97-3.06)†	1.39 (0.77-2.49)	1.40 (0.77-2.54)
Employment pattern (relisted)				
Stable[f]	1,931	1.00	1.00	1.00
Unstable[g]	572	1.72 (1.36-2.17)*	1.56 (1.23-1.98)*	1.52 (1.19-1.95)*
Inexperienced	69	1.77 (1.00-3.13)†	1.44 (0.81-2.58)	1.45 (0.80-2.63)
Workplace size for the longest held job				
≥300 employees	1,211	1.00	1.00	1.00
50–299 employees	484	1.07 (0.79-1.44)	1.02 (0.75-1.38)	1.00 (0.74-1.37)
10–49 employees	317	1.73 (1.26-2.36)*	1.59 (1.16-2.18)*	1.56 (1.12-2.17)*
1–9 employees	491	1.67 (1.27-2.19)*	1.57 (1.19-2.07)*	1.51 (1.13-2.02)*
Inexperienced	69	1.87 (1.05-3.36)*	1.51 (0.84-2.73)	1.52 (0.83-2.77)
Workplace size (relisted)				
Large (≥50 employees)	1,695	1.00	1.00	1.00
Small (1–49 employees)	808	1.66 (1.34-2.06)*	1.57 (1.26-1.96)*	1.53 (1.21-1.93)*
Inexperienced	69	1.84 (1.03-3.27)*	1.50 (0.84-2.70)	1.52 (0.83-2.75)

† $p < 0.10$, * $p < 0.05$.

[a]Adjusted for age.

[b]Adjusted for age, marital status, education, self-perceived economic status, body mass index, chronic illnesses, smoking, alcohol consumption, sports habits, cognitive functioning, and depression.

[c]White collar: managers, professionals, and technicians.

[d]Pink collar: clerical, sales, and services workers.

[e]Blue collar: manufacturing, transport, machine, construction, mining, protective services, agricultural, forestry, fishery, carrying, cleaning, and packaging workers.

[f]Stable: full-time private sector workers and civil servants.

[g]Unstable: non-regular employees, self-employed/freelancers, and others.

eTable 3. Odds ratios for decline in instrumental activities of daily living (95% confidence intervals) by work history (n=3,285 women)

	n	Unadjusted	Model 1[a]	Model 2[b]
Working status				
Retired	2,464	1.00	1.00	1.00
Working	486	0.37 (0.23-0.62)*	0.63 (0.37-1.06)†	0.71 (0.41-1.22)
Inexperienced	335	1.40 (0.98-2.00)†	1.10 (0.75-1.61)	1.18 (0.79-1.76)
Total working years				
≥25 years	1,199	1.00	1.00	1.00
15–24 years	592	0.89 (0.60-1.30)	0.94 (0.63-1.40)	0.97 (0.64-1.48)
5–14 years	767	1.06 (0.76-1.49)	1.21 (0.85-1.72)	1.20 (0.83-1.75)
1–4 years	392	1.28 (0.86-1.90)	1.26 (0.83-1.93)	1.16 (0.74-1.81)
0 years (inexperienced)	335	1.61 (1.09-2.39)*	1.23 (0.80-1.87)	1.30 (0.83-2.02)
Total working years (relisted)				
≥25 years	1,199	1.00	1.00	1.00
1–24 years	1,751	1.05 (0.80-1.38)	1.13 (0.84-1.50)	1.11 (0.82-1.51)
0 years (inexperienced)	335	1.61 (1.09-2.39)*	1.23 (0.80-1.87)	1.29 (0.83-2.01)
Occupation for the longest held job				
Managers & professionals & technicians	466	1.00	1.00	1.00
Clerical workers	950	0.92 (0.59-1.42)	1.05 (0.66-1.66)	0.94 (0.57-1.55)
Sales workers	311	1.00 (0.57-1.75)	1.21 (0.67-2.18)	0.97 (0.52-1.81)
Services workers	339	0.82 (0.46-1.46)	1.11 (0.61-2.05)	0.79 (0.41-1.50)
Manufacturing workers	579	1.49 (0.95-2.32)†	1.73 (1.08-2.78)*	1.20 (0.72-2.01)
Carrying, cleaning, and packaging workers	73	1.62 (0.72-3.65)	1.67 (0.69-4.04)	1.12 (0.45-2.81)
Agricultural, forestry, fishery, transport, machine, construction, and mining workers	27	1.05 (0.24-4.63)	0.76 (0.16-3.59)	0.90 (0.19-4.34)
Others	205	2.16 (1.27-3.67)*	1.68 (0.96-2.96)†	1.06 (0.58-1.94)
Inexperienced	335	1.78 (1.10-2.89)*	1.44 (0.86-2.41)	1.22 (0.71-2.10)
Occupation (relisted)				
White collar[c]	466	1.00	1.00	1.00
Pink collar[d]	1,600	0.91 (0.61-1.37)	1.09 (0.71-1.67)	0.91 (0.57-1.45)
Blue collar[e]	679	1.48 (0.96-2.29)†	1.67 (1.05-2.64)*	1.18 (0.72-1.94)
Others	205	2.16 (1.27-3.67)*	1.68 (0.96-2.96)†	1.06 (0.58-1.94)
Inexperienced	335	1.78 (1.10-2.89)*	1.44 (0.86-2.41)	1.21 (0.70-2.10)

eTable 3. Continued

	n	Unadjusted	Model 1[a]	Model 2[b]
Employment pattern for the longest held job				
Full-time private sector workers	997	1.00	1.00	1.00
Civil servants	261	1.12 (0.68-1.87)	0.81 (0.47-1.38)	0.87 (0.50-1.55)
Non-regular employees	774	0.63 (0.42-0.95)*	0.85 (0.55-1.32)	0.71 (0.46-1.12)
Self-employed/freelancers	729	1.70 (1.22-2.36)*	1.33 (0.94-1.90)	1.26 (0.87-1.82)
Others	189	1.61 (0.96-2.68)†	1.35 (0.78-2.34)	1.21 (0.68-2.15)
Inexperienced	335	1.74 (1.16-2.62)*	1.23 (0.79-1.90)	1.24 (0.78-1.95)
Employment pattern (relisted)				
Stable[f]	1,258	1.00	1.00	1.00
Unstable[g]	1,692	1.15 (0.87-1.51)	1.22 (0.92-1.64)	1.08 (0.80-1.46)
Inexperienced	335	1.70 (1.15-2.51)*	1.29 (0.84-1.96)	1.27 (0.82-1.96)
Workplace size for the longest held job				
≥300 employees	703	1.00	1.00	1.00
50–299 employees	562	0.77 (0.48-1.23)	0.82 (0.50-1.35)	0.67 (0.40-1.13)
10–49 employees	637	1.26 (0.84-1.89)	1.24 (0.81-1.91)	1.07 (0.68-1.68)
1–-9 employees	1,048	1.49 (1.04-2.12)*	1.31 (0.89-1.91)	1.08 (0.72-1.61)
Inexperienced	335	1.85 (1.19-2.87)*	1.30 (0.81-2.09)	1.20 (0.73-1.96)
Workplace size (relisted)				
Large (≥50 employees)	1,265	1.00	1.00	1.00
Small (1–49 employees)	1,685	1.56 (1.18-2.07)*	1.39 (1.03-1.88)*	1.28 (0.94-1.75)
Inexperienced	335	2.06 (1.38-3.08)*	1.41 (0.92-2.16)	1.42 (0.90-2.22)

† p <0.10, * p <0.05.

[a] Adjusted for age.

[b] Adjusted for age, marital status, education, self-perceived economic status, body mass index, chronic illnesses, smoking, alcohol consumption, sports habits, cognitive functioning, and depression.

[c] White collar: managers, professionals, and technicians.

[d] Pink collar: clerical, sales, and services workers.

[e] Blue collar: manufacturing, transport, machine, construction, mining, protective services, agricultural, forestry, fishery, carrying, cleaning, and packaging workers.

[f] Stable: full-time private sector workers and civil servants.

[g] Unstable: non-regular employees, self-employed/freelancers, and others.

eTable 4. Results of consideration for multicollinearity

	Variance Inflation Factors		
	All (n=5,857)	Men (n=2,572)	Women (n=3,285)
Working status at baseline	1.50	1.30	2.06
Total working years	1.63	1.71	1.48
Occupation for the longest held job	1.97	1.46	2.21
Employment pattern for the longest held job	2.75	2.56	2.54
Workplace size for the longest held job	2.52	2.25	2.42

eTable 5. Sensitivity analyses between imputed and complete case datasets

	Men		Women	
	MI data<br>n=2,572<br>OR[a] (95% CI)	Complete data<br>n=2,086<br>OR[a] (95% CI)	MI data<br>n=3,285<br>OR[a] (95% CI)	Complete data<br>n=2,509<br>OR[a] (95% CI)
Working status at baseline				
Retired	1.00	1.00	1.00	1.00
Working	0.96 (0.74-1.24)	1.00 (0.75-1.33)	0.71 (0.41-1.22)	0.53 (0.26-1.09)
Inexperienced	1.28 (0.71-2.31)	1.72 (0.90-3.30)	1.18 (0.79-1.76)	1.06 (0.65-1.74)
Total working years				
≥25	1.00	1.00	1.00	1.00
1–24	1.22 (0.76-1.97)	1.16 (0.64-2.10)	1.11 (0.82-1.51)	0.91 (0.64-1.31)
0 (inexperienced)	1.30 (0.72-2.35)	1.74 (0.91-3.32)	1.29 (0.83-2.01)	1.06 (0.62-1.81)
Occupation for the longest held job				
White collar	1.00	1.00	1.00	1.00
Pink collar	1.06 (0.80-1.38)	1.07 (0.80-1.45)	0.91 (0.57-1.45)	1.02 (0.59-1.75)
Blue collar	1.19 (0.89-1.60)	1.13 (0.81-1.58)	1.18 (0.72-1.94)	1.35 (0.75-2.43)
Others	0.73 (0.36-1.49)	0.56 (0.21-1.47)	1.06 (0.58-1.94)	1.02 (0.46-2.28)
Inexperienced	1.35 (0.74-2.47)	1.79 (0.92-3.48)	1.21 (0.70-2.10)	1.21 (0.63-2.33)
Employment pattern for the longest held job				
Stable	1.00	1.00	1.00	1.00
Unstable	1.52 (1.19-1.95)	1.50 (1.13-2.00)	1.08 (0.80-1.46)	0.92 (0.64-1.32)
Inexperienced	1.45 (0.80-2.63)	1.92 (1.00-3.69)	1.27 (0.82-1.96)	1.07 (0.63-1.81)
Workplace size for the longest held job				
Large	1.00	1.00	1.00	1.00
Small	1.53 (1.21-1.93)	1.61 (1.23-2.09)	1.28 (0.94-1.75)	1.09 (0.76-1.56)
Inexperienced	1.52 (0.83-2.75)	2.06 (1.07-3.97)	1.42 (0.90-2.22)	1.17 (0.69-2.00)

CI, confidence interval; MI, multiple imputation; OR, odds ratio.

[a]Adjusted for age, marital status, educational background, self-perceived economic status, body mass index, chronic illnesses, smoking, alcohol consumption, sports habits, cognitive functioning, and depression.
